# GLORIA - A globally representative hyperspectral *in situ* dataset for optical sensing of water quality

**DOI:** 10.1038/s41597-023-01973-y

**Published:** 2023-02-16

**Authors:** Moritz K. Lehmann, Daniela Gurlin, Nima Pahlevan, Krista Alikas, Ted Conroy, Janet Anstee, Sundarabalan V. Balasubramanian, Cláudio C. F. Barbosa, Caren Binding, Astrid Bracher, Mariano Bresciani, Ashley Burtner, Zhigang Cao, Arnold G. Dekker, Courtney Di Vittorio, Nathan Drayson, Reagan M. Errera, Virginia Fernandez, Dariusz Ficek, Cédric G. Fichot, Peter Gege, Claudia Giardino, Anatoly A. Gitelson, Steven R. Greb, Hayden Henderson, Hiroto Higa, Abolfazl Irani Rahaghi, Cédric Jamet, Dalin Jiang, Thomas Jordan, Kersti Kangro, Jeremy A. Kravitz, Arne S. Kristoffersen, Raphael Kudela, Lin Li, Martin Ligi, Hubert Loisel, Steven Lohrenz, Ronghua Ma, Daniel A. Maciel, Tim J. Malthus, Bunkei Matsushita, Mark Matthews, Camille Minaudo, Deepak R. Mishra, Sachidananda Mishra, Tim Moore, Wesley J. Moses, Hà Nguyễn, Evlyn M. L. M. Novo, Stéfani Novoa, Daniel Odermatt, David M. O’Donnell, Leif G. Olmanson, Michael Ondrusek, Natascha Oppelt, Sylvain Ouillon, Waterloo Pereira Filho, Stefan Plattner, Antonio Ruiz Verdú, Salem I. Salem, John F. Schalles, Stefan G. H. Simis, Eko Siswanto, Brandon Smith, Ian Somlai-Schweiger, Mariana A. Soppa, Evangelos Spyrakos, Elinor Tessin, Hendrik J. van der Woerd, Andrea Vander Woude, Ryan A. Vandermeulen, Vincent Vantrepotte, Marcel R. Wernand, Mortimer Werther, Kyana Young, Linwei Yue

**Affiliations:** 1 Xerra Earth Observation Institute, PO Box 400, Alexandra, 9340 New Zealand; 2 School of Science, University of Waikato, Private Bag 3105, Hamilton, 3240 New Zealand; 3 Wisconsin Department of Natural Resources, Bureau of Water Quality, 101 S Webster Street, Madison, WI 53707 USA; 4 Science Systems and Applications, Inc. (SSAI), Lanham, MD USA; 5 NASA Goddard Space Flight Center, Greenbelt, MD USA; 6 Tartu Observatory of the University of Tartu, Tartumaa, 61602 Estonia; 7 Coasts and Oceans Systems Program (COS), CSIRO Environment Business Unit, Acton, ACT 2601 Australia; 8 GeoSensing and Imaging Consultancy, Trivandrum, Kerala India; 9 Instrumentation Lab for Aquatic Systems (LabISA), National Institute for Space Research (INPE), São José dos Campos, Brazil; 10 Environment and Climate Change Canada, Burlington, ON Canada; 11 Phytooptics Group, Physical Oceanography of Polar Seas, Climate Sciences, Alfred Wegener Institute, Helmholtz Centre for Polar and Marine Research, Bremerhaven, Germany; 12 Department of Physics and Electrical Engineering, Institute of Environmental Physics, University of Bremen, Bremen, Germany; 13 National Research Council of Italy, Institute for Electromagnetic Sensing of the Environment, CNR-IREA, Milano, Italy; 14 Cooperative Institute for Great Lakes Research, University of Michigan, 4840 South State Road, Ann Arbor, MI 48108 USA; 15 Nanjing Institute of Geography and Limnology, Chinese Academy of Sciences, Nanjing, 210008 China; 16 SatDek Pty Ltd, 99 Read Rd, Sutton, NSW 2620 Australia; 17 Wake Forest University, Engineering, 455 Vine Street, Winston-Salem, NC 27101 USA; 18 NOAA Great Lakes Environmental Research Laboratory, Ann Arbor, MI USA; 19 Department of Geography, Universidad de la República, Montevideo, Uruguay; 20 Institute of Biology and Earth Sciences, Pomeranian University, Arciszewskiego 22, 76-200 Slupsk, Poland; 21 Department of Earth and Environment, Boston University, Boston, MA USA; 22 German Aerospace Center (DLR), Remote Sensing Technology Institute, Wessling, Germany; 23 University of Nebraska-Lincoln, School of Natural Resources, 3310 Holdrege Street, Lincoln, NE 68503 USA; 24 University of Wisconsin-Madison, Aquatic Sciences Center, 1975 Willow Drive, Madison, WI 53706 USA; 25 Michigan Technological University, Great Lakes Research Center, 100 Phoenix Drive, Houghton, MI 49931 USA; 26 Faculty of Urban Innovation, Yokohama National University, Tokiwadai 79-5, Hodogaya, Yokohama, Kanagawa Japan; 27 Swiss Federal Institute of Aquatic Science and Technology, Department of Surface Waters – Research and Management, Dübendorf, Switzerland; 28 Université du Littoral Côte d’Opale, CNRS, Univ. Lille, IRD, UMR 8187 - LOG - Laboratoire d’Océanologie et de Géosciences, F-62930 Wimereux, France; 29 Earth and Planetary Observation Sciences (EPOS), Biological and Environmental Sciences, Faculty of Natural Sciences, University of Stirling, Stirling, UK; 30 Plymouth Marine Laboratory, Plymouth, PL1 3DH UK; 31 NASA Ames Research Center, Moffett Field, CA USA; 32 Department of Physics and Technology, University of Bergen, Bergen, Norway; 33 University of California-Santa Cruz, Ocean Sciences Department, Institute of Marine Sciences, 1156 High Street, Santa Cruz, CA 95064 USA; 34 Department of Earth Sciences, Indiana University-Purdue University, Indianapolis, IN USA; 35 University of Massachusetts-Dartmouth, School for Marine Science and Technology West, 706 South Rodney French Blvd., New Bedford, MA 02744 USA; 36 Coasts and Oceans Systems Program (COS), CSIRO Environment Business Unit, Ecosciences Precinct, 41 Boggo Road, Dutton Park, QLD 4102 Australia; 37 Faculty of Life and Environmental Sciences, University of Tsukuba, Ibaraki, Japan; 38 CyanoLakes (Pty) Ltd, Sydney, 2126 Australia; 39 Departament de Biologia Evolutiva, Ecologia i Ciències Ambientals, Facultat de Biologia, Universitat de Barcelona, Av. Diagonal 643, 08028 Barcelona, Spain; 40 Department of Geography, University of Georgia, Athens, GA 30602 USA; 41 National Centers for Coastal Ocean Science, National Oceanic and Atmospheric Administration, 1305 East-West Hwy, Silver Spring, MD 20910 USA; 42 Harbor Branch Oceanographic Institute, Florida Atlantic University, Fort Pierce, FL USA; 43 U.S. Naval Research Laboratory, 4555 Overlook Ave SW, Washington, DC 20375 USA; 44 Faculty of Geology, VNU University of Science, Ha Noi, Vietnam; 45 Royal Netherlands Institute for Sea Research, Physical Oceanography, Marine Optics & Remote Sensing, Den Burg, Texel, Netherlands; 46 Upstate Freshwater Institute, Syracuse, NY USA; 47 Department of Forest Resources, University of Minnesota, St. Paul, MN USA; 48 NOAA Center for Satellite Applications and Research, College Park, MD USA; 49 Earth Observation and Modelling, Kiel University, Department of Geography, 24118 Kiel, Germany; 50 UMR LEGOS, University of Toulouse, IRD, CNES, CNRS, UPS, 14 Avenue Edouard Belin, 31400 Toulouse, France; 51 Department Water-Environment-Oceanography, University of Science and Technology of Hanoi (USTH), Vietnamese Academy of Science and Technology (VAST), 18 Hoang Quoc Viet, Hanoi, 100000 Vietnam; 52 Department of Geosciences, Federal University of Santa Maria, Av. Roraima, 1000, 97105-900 Santa Maria, Rio Grande do Sul Brazil; 53 Laboratory for Earth Observation, University of Valencia, Catedrático Agustín Escardino 9, Paterna (Valencia), 46980 Spain; 54 Faculty of Engineering, Kyoto University of Advanced Science (KUAS), 18 Yamanouchi Gotanda, Ukyo, Kyoto, Japan; 55 Creighton University, Department of Biology, Omaha, NE 68178 USA; 56 Japan Agency for Marine-Earth Science and Technology (JAMSTEC), Showa-machi 3173-25, Yokohama, Kanagawa 2360001 Japan; 57 Department of Water & Climate Risk, Institute for Environmental Studies (IVM), Vrije Universiteit, Amsterdam, Netherlands; 58 Ocean Ecology Laboratory, NASA Goddard Space Flight Center, Greenbelt, MD USA; 59 China University of Geosciences, School of Geography and Information Engineering, Wuhan, China

**Keywords:** Limnology, Environmental impact

## Abstract

The development of algorithms for remote sensing of water quality (RSWQ) requires a large amount of *in situ* data to account for the bio-geo-optical diversity of inland and coastal waters. The GLObal Reflectance community dataset for Imaging and optical sensing of Aquatic environments (GLORIA) includes 7,572 curated hyperspectral remote sensing reflectance measurements at 1 nm intervals within the 350 to 900 nm wavelength range. In addition, at least one co-located water quality measurement of chlorophyll *a*, total suspended solids, absorption by dissolved substances, and Secchi depth, is provided. The data were contributed by researchers affiliated with 59 institutions worldwide and come from 450 different water bodies, making GLORIA the *de-facto* state of knowledge of *in situ* coastal and inland aquatic optical diversity. Each measurement is documented with comprehensive methodological details, allowing users to evaluate fitness-for-purpose, and providing a reference for practitioners planning similar measurements. We provide open and free access to this dataset with the goal of enabling scientific and technological advancement towards operational regional and global RSWQ monitoring.

## Background & Summary

Light from the sun reflected back across the water-air interface carries characteristic spectral signatures of several key water quality constituents due to their wavelength-specific absorption and scattering properties^1,2^. Chlorophyll *a*, total suspended solids, and colored dissolved organic matter are the dominant optically active constituents in inland and coastal waters^3,4^, and common measures of water quality used for the management of ecosystem and public health^5–8^. Accurate measurements of spectral reflectance (i.e., the upwelling radiance normalized by the downwelling solar irradiance) are the foundation for synoptic and cost-effective environmental monitoring applications using satellite sensors, automated sensors installed near the water surface and portable instruments for manual field surveys^9^.

Space-borne instruments have been providing accurate estimates of chlorophyll *a* and particle backscattering in the open ocean since the late 1990s with data from the Sea-viewing Wide Field-of-view Sensor (SeaWiFS) followed by many others, including the MEdium Resolution Imaging Spectrometer (MERIS) and Moderate Resolution Imaging Spectroradiometer (MODIS) in the 2000s, and the Ocean and Land Colour Instrument (OLCI) and Visible Infrared Imaging Radiometer Suite (VIIRS) over the last decade^10–17^. However, in coastal and inland waters, uncertainties in these estimates are typically much higher due to factors that include diverse atmospheric contributions, stray light from adjacent land areas, potentially uncorrelated variability of optically active constituents, and, in optically shallow water, bottom reflection^9,18–20^. Further, coarse-resolution imagers with a nominal resolution near 1 km are limited in nearshore and narrow systems where modern high-resolution missions like Landsat-8 and Sentinel-2 offer valid observations^21^. Overall, the retrieval of water quality in lakes, rivers, estuaries, lagoons and nearshore coastal waters remains an active area of research where improvements are needed so that satellite observations can fulfill their potential and become part of routine monitoring programs for ecosystem states, trends, and public-health alerting systems^22–26^.

Large and globally representative *in situ* datasets are essential for the development and validation of bio-optical algorithms to support large-scale monitoring using satellite Earth observation technologies. Such datasets are particularly scarce and geographically fragmented from inland and coastal waters as radiometric measurements are not part of most routine sampling programs and many lakes are remote and difficult to access.

We address these shortcomings with our GLObal Reflectance community dataset for Imaging and optical sensing of Aquatic environments (GLORIA). GLORIA includes over 7000 curated hyperspectral remote sensing reflectance (*R*_rs_, sr^−1^) and co-located chlorophyll *a* (Chl*a*, mg m^−3^), total suspended solids (TSS, g m^−3^), absorption by colored dissolved organic matter (CDOM) at 440 nm wavelength (*a*_CDOM_(440), m^−1^) and Secchi depth (m) measurements. The data were contributed by researchers affiliated with 59 institutions in 20 countries who made the measurements for a range of objectives under diverse funding sources and resource levels, but shared attention to strict sampling protocols, tenacity to reach remote and inaccessible sites, commitment to establish long-term trend monitoring sites, and the recognition of the value of open-access datasets for public benefit. With its almost global coverage, geomorphic range of water bodies, and 30-year time span (Fig. 2), GLORIA represents the *de-facto* state of knowledge of *in situ* coastal and inland water bio-geo-optical diversity. Subsets of the data have already produced significant contributions to global algorithm development for the satellite-based estimation of Chl*a*, TSS, and *a*_CDOM_(440) using data-intensive machine-learning methods^27–31^ or global semi-analytical approaches^32^. Where they were available, we also provide uncertainty estimates of *R*_rs_ and water quality measurements as standard deviations and means from replicate measurements. Nevertheless, some methodological detail which is currently considered relevant may not have been recorded at the time of observation, which limits our ability to retrospectively assess sources of uncertainty to subsets of the global dataset.

GLORIA builds upon the existing data repositories aimed at remote sensing studies of aquatic environments. We address poorly represented optically complex coastal and inland waters in existing open-data platforms such as the SeaWiFS Bio-optical Archive and Storage System (SeaBASS, https://seabass.gsfc.nasa.gov)^33,34^. In contrast to other relevant data repositories, such as the Lake Bio-optical Measurements and Matchup Data for Remote Sensing (LIMNADES, https://limnades.stir.ac.uk) database, GLORIA is open-access. By carrying out consistent quality control across the entire dataset, and providing comprehensive methodological details associated with each measurement, we have produced an analysis-ready, standalone data package for the community.

The commitment of space agencies towards maintaining and enhancing optical Earth observing systems and the burgeoning fleet of commercial platforms indicate that our coupled reflectance-water quality attribute dataset fills a strong need to facilitate algorithm and application development. We anticipate that our collection of field setups and methodologies will encourage targeted data collection for the calibration and validation of upcoming satellite sensors^35–37^, as well as the growth of *in situ* observatories^38–40^.

## Methods

The GLORIA dataset was collated from the aquatic optics community of researchers or research groups working towards a range of goals including the routine monitoring of high-priority sites, one-off bio-optical characterization of a range of water bodies, data gathering to support algorithm development, or designated sampling for validating equivalent satellite-derived products. Efforts to gather this data started in 2018 with the second atmospheric correction intercomparison exercise (ACIX-II Aqua), an international collaboration to test processors that generate aquatic reflectance products from radiance measurements made at the top of the atmosphere^19^. Requests for contributions were made at pertinent conference sessions and via the research networks of individuals. These requests were for quality assured remote sensing reflectance spectra at 1 nm intervals within the 350 to 900 nm wavelength range and at least one co-located water quality attribute (Chl*a*, TSS, *a*_CDOM_(440), or Secchi depth), and associated uncertainties. The sections below provide more details of the data and processing.

### Radiometric data collection and processing

**Fig. 1:**
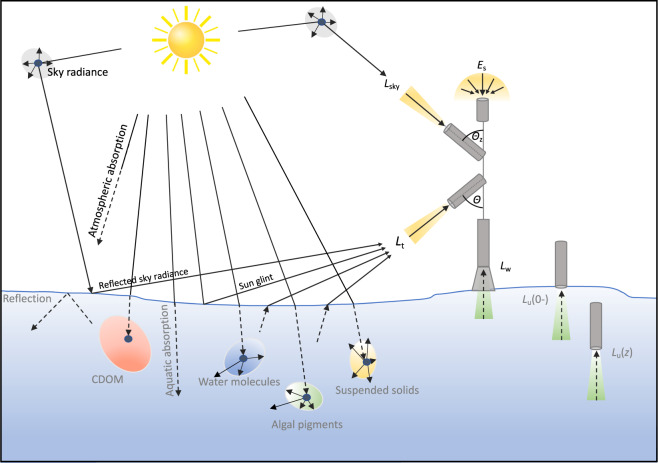
Optical processes of absorption and scattering in the atmosphere and the water determine the amount and spectral nature of light received by a sensor. Remote sensing reflectance, the central radiometric quantity of the GLORIA dataset, is the ratio of the water-leaving radiance just above the water surface (*L*_w_) over above-water downwelling irradiance (*E*_s_).

The central radiometric quantity reported in our dataset is remote sensing reflectance, *R*_rs_ (sr^−1^). It is defined as the ratio of the water-leaving radiance just above the water surface (*L*_w_(0+), W m^−2^ sr^−1^ nm^−1^) over above-water downwelling irradiance (*E*_s_, W m^−2^ nm^−1^)(Eq. 1, Fig. 1). We use the symbology of Ruddick *et al*.^41^ with slight modifications:

1 $${R}_{{\rm{rs}}}(\lambda ,\Theta ,\phi )=\frac{{L}_{{\rm{w}}}(\lambda ,\Theta ,\phi )}{{E}_{{\rm{s}}}(\lambda )}$$

*R*_rs_ and *L*_w_ are dependent on the viewing nadir angle *Θ* (measured from the downward vertical axis) and azimuth viewing angle *ɸ* (measured clockwise from the sun); *λ* identifies the wavelength dependence. For aquatic remote sensing applications, it is conventional to define *R*_rs_ as derived from a sensor looking straight down *L*_w_(*λ*, *Θ* = 0) where *ɸ* is not defined^42^. Therefore, we omit *λ*, *Θ*, and *ɸ* for notational brevity. Several methods and instruments were used for the measurement of the downwelling and upwelling radiometric quantities reported in our dataset. Here we provide brief descriptions of the broad types of methodologies used for their measurement, and a list at the end of this section gives a formal summary.

*L*_w_ can be measured directly using a radiometer just above the water surface, looking vertically down and shielded from light reflected off the water surface^43^. Other common techniques include measurement of the upwelling radiance at nadir below the water surface (*L*_u_(0-))^44^, or from above the water surface where the sensor is directed at a non-zero nadir angle (*L*_t_)^45^. Both of these radiance measurements require conversions to *L*_w_, which are referenced in the list at the end of this section. In brief, *L*_u_(0-) can be derived by extrapolating upwelling radiance from measurements at practical depths below the water surface to just below the water surface^46^. Propagation through the water-air interface by accounting for the reduction of radiance by internal reflection off the water surface yields *L*_w_. The estimation of *L*_w_ from *L*_t_ is more involved, as *L*_t_ contains a considerable amount of sky radiance reflected off the water surface into the sensor field of view (reflected sky radiance) in addition to *L*_w_(*Θ*, *ɸ*), where we note the angular dependence to emphasize the need for conversion to *Θ* = 0. Sky radiance (*L*_sky_) is therefore usually measured simultaneously with *L*_t_ at the same azimuth angles and at zenith angles *Θ*_z_ (from the upward vertical axis) near 40°^42^.

Three different approaches were used to measure *E*_s_ in the present dataset and a detailed review is provided by Ruddick *at al*.^47^. Most commonly *E*_s_ was measured directly using a plane irradiance sensor above the water surface directed straight upwards. The second most used method employed a downward pointing radiance sensor measuring the reflectance of a horizontally held Lambertian plaque with known reflective properties. This method has the advantage that a single sensor can be used for all measurements needed for the calculation of *R*_rs_, potentially reducing cost, equipment load and uncertainties from the intercalibration of several sensors. In some cases, *E*_s_ was estimated from irradiance measurements below the water surface (just below the surface: *E*_d_(0-), or at depth *z*: *E*_d_(*z*)). These measurements are typical of autonomous installations on vertical sensor chains or a single sensor package on a vertically profiling platform^44^.

The instruments used for the radiometric measurements for each entry of the GLORIA dataset are part of the metadata (file *GLORIA_meta_and_lab.csv*) and are provided in the list at the end of this section. These include those customarily used for validation of satellite-derived aquatic reflectance, such as RAMSES (TriOS, Germany), HyperOCR (manufactured by Sea-Bird Scientific, USA; previously manufactured by Satlantic Inc., Canada) and C-OPS (Biospherical Instruments Inc., USA). The RAMSES and HyperOCR have 256 channel silicon photodiode array detectors with a 10 nm spectral resolution and a spectral sampling of 3.3 nm per pixel. The typical setup for RAMSES instruments for our dataset is an above-surface installation with a vertical *E*_s_ sensor and *L*_sky_ and *L*_t_ sensors at 40–42° zenith and nadir angles, respectively (Fig. 1). HyperOCR instruments are typically installed on a floating frame to measure *E*_s_, and *L*_u_ or *L*_w_ at zero nadir angle while the HyperPRO (and HyperPro II) are free-falling setups of the HyperOCR designed to measure vertical profiles in the water column. The C-OPS configuration is similar to the HyperOCR, but the instrument only has 19 spectral bands of 10 nm width. The HyperSAS is a three-sensor setup of the HyperOCR for above-surface installation on structures overlooking the water or ships, similar to the RAMSES setup. The Water Insight WISP-3 is a self-contained handheld unit with optical inputs for *E*_s_, *L*_sky_ and *L*_t_ leading to separate spectrometers^48^.

A number of instruments used accommodate a single optical input into handheld units or portable instruments and need to be pointed to provide the different radiometric measurements (ASD FieldSpec range, Satlantic HyperGun, Spectra Vista, Spectral Evolution, Spectron Engineering and Photo Research SpectraScan devices).

Some investigators integrated compact spectrometers (manufactured by Ocean Insight, Inc., formerly known as Ocean Optics, Inc., USA) with data loggers and optical fibers on frames or poles that can be pointed away from observation platforms. Measurements would either be accomplished through several instruments and optical fibers oriented for the respective radiometric quantities, or a single sequentially reoriented fiber.

Data contributors provided radiometric measurements interpolated to 1 nm intervals over the 350 to 900 nm wavelength range. The instrument-specific bandwidths of the original measurements are provided in the data table (file *GLORIA_meta_and_lab.csv*, column ‘Spectral_resolution_nm’). Due to instrument and processing constraints, some spectra span the range from 400 to 750 nm, or nearby bounds. The radiometric data for each GLORIA entry may be from a single measurement, or the mean or median of several measurements over a time interval. When available, the data contributors provided the spectral *R*_rs_ means, standard deviations, and numbers of measurements for sampling events. Quality control was conducted on all received spectra (see section *Technical validation*).

The measurement setups and instruments used for radiometric measurements are listed below. The number of the method corresponds to the column ‘Measurement_method’ in *GLORIA_meta_and_lab.csv*. References to published descriptions of the approach and applications are provided where available.

1. **Sequential** ***L***_**t**_, ***L***_**sky**_**, and** ***E***_**s**_ **via a plaque on MP (moving platform)**
  Instruments: ASD FieldSpec, Photo Research PR-650 SpectraScan Colorimeter, Sea-Bird Scientific/Satlantic HyperGun, Spectra Vista GER1500, Spectral Evolution SR-3500/PSR-1100f, Spectron Engineering SE-590, TriOS RAMSES
  Approach: Mobley^45^
  Applications: Bresciani *et al*.^49^; Kudela *et al*.^50^; Zolfaghari *et al*.^51^
2. ***L***_**t**_**, L**_**sky**_**, and** ***E***_**s**_ **on MP**
  Instruments: Water Insight WISP-3
  Approach: Mobley^45^
  Applications: Hommersom *et al*.^48^
3. ***L***_**u**_**(0-) and** ***E***_**s**_ **on pole connected to a spectrometer via fiber optics from MP or water edge**
  Instruments: Ocean Insight/Ocean Optics USB2000/USB2000 + /USB4000
  Approach: Chipman *et al*.^52^
  Applications: Gurlin *et al*.^53^; Schalles and Hladik^54^; Li *et al*.^55^; Mishra *et al*.^56^; Brezonik *et al*.^57^; Werther *et al*.^58^
4. ***L***_**w**_**(0+) skylight blocked and** ***E***_**s**_ **afloat away from MP**
  Instruments: Sea-Bird Scientific/Satlantic HyperOCR
  Approach: Lee *et al*.^55^
  Applications: Wei *et al*.^59^
5. ***L***_**u**_**(0-) afloat away from MP**, ***E***_**s**_ **on MP**
  Instruments: Sea-Bird Scientific/Satlantic HyperOCR, TriOS RAMSES
6. ***L***_**t**_, ***L***_**sky**_**, and** ***E***_**s**_ **on MP**
  Instruments: Sea-Bird Scientific/Satlantic HyperSAS, TriOS RAMSES
  Approach: Mobley^45^; Simis and Olsson^60^
  Applications: Qin *et al*.^61^; Warren *et al*.^62^
7. ***L***_**t**_, ***L***_**sky**_**, and** ***E***_**s**_ **on a frame deployed on MP**
  Instruments: TriOS RAMSES
  Approach: Mobley^45^; Mobley^63^
  Applications: Maciel *et al*.^64^; Cairo *et al*.^65^; da Silva *et al*.^66^
8. ***L***_**u**_**(0-) and** ***E***_**d**_**(0-) in-water profiling from MP**, ***E***_**s**_ **on MP**
  Instruments: Biospherical C-OPS, Sea-Bird Scientific/Satlantic HyperOCR, TriOS RAMSES
  Approach: Mueller *et al*.^44^; Lubac and Loisel^67^
  Applications: Binding *et al*.^68^
9. ***L***_**u**_**(0-) and** ***E***_**d**_**(*z*) units on a depth adjustable bar (measurements at −0.21 and −0.67 m) on a frame afloat away from MP**, ***E***_**s**_ **unit lifted above water surface for** ***E***_**s**_
  Instruments: TriOS RAMSES
  Approach: Fritz *et al*.^69^
10. ***L***_**u**_**(0-) and** ***E***_**d**_**(0-) from winch on MP**, ***E***_**s**_ **on MP**
  Instruments: TriOS RAMSES
  Approach: Zibordi and Talone^70^
11. ***L***_**t**_ **and** ***E***_**s**_ **on pole from water edge**
  Instruments: TriOS RAMSES
  Approach: Kutser *et al*.^71^
12. ***L***_**u**_**(0-) and** ***E***_**d**_**(0-) autonomous in-water profiling from a fixed platform**
  Instruments: Sea-Bird Scientific/Satlantic HyperOCR
  Approach: Mueller *et al*.^44^
  Applications: Minaudo *et al*.^72^
13. **Sequential** ***L***_**t**_ **and** ***E***_**s**_ **via a plaque, mounted on gimbal stabilized pole from MP**
  Instruments: Ocean Insight/Ocean Optics STS-VIS
14. ***L***_**u**_**(0-) (and** ***E***_**d**_**(0-) only for depth information) from in-water profiling from MP**, ***E***_**s**_ **recorded simultaneously from same MP very close to profiler deployment**
  Instruments: TriOS RAMSES
  Approach: Mueller *et al*.^44^; Stramski *et al*.^73^
  Applications: Bracher *et al*.^74^; Tilstone *et al*.^75^
15. ***L***_**t**_, ***L***_**sky**_, ***E***_**s**_**, combined with one** ***L***_**u**_ **unit (aperture at −0.05 to −0.10 m) placed on a pole**
  Instruments: TriOS RAMSES
16. **Sequential** ***L***_**u**_**(0-) and** ***E***_**s**_ **via a plaque, both measurements using an optical fiber to a black masked perspex tube**
  Instruments: Spectron Engineering SE-590
  Approach: Dekker^76^
17. ***L***_**u**_**(0-) and** ***E***_**d**_**(z) units on a floating frame (measurements at −0.4 m (*L***_**u**_**) and −0.1 m (*E***_**d**_**)) drifting 10 m away from vessel**

Instruments: TriOS RAMSES

Approach: Fritz *et al*.^69^

### SeaBASS data

GLORIA includes approximately 1100 entries from SeaBASS^33^. We searched SeaBASS for reflectance spectra with concomitant water quality measurements and ensured that these are from inland and coastal waters only by mapping sampling locations of all records from water depths less than 200 m. Where water depth was not part of the SeaBASS record, we assigned it based on the General Bathymetric Chart of the Ocean (GEBCO_2021 Grid sub-ice topo/bathy)^77^. Several metadata fields were unavailable for this data, but SeaBASS dataset identifiers are provided to allow further research if needed. All SeaBASS data were included in our quality control process. While SeaBASS allows the upload of uncertainty data for radiometry and water quality, the entries we located for inland and coastal waters did not contain this information.

### Water sample analysis

Water quality attributes Chl*a*, TSS and *a*_CDOM_(440) were determined using well established high-accuracy laboratory methods. The method for each analysis is identified in the columns ‘Chl_method’, ‘TSS_method’, and ‘aCDOM_method’ in the file *GLORIA_meta_and_lab.csv* and method details are provided in *GLORIA_variables_and_methods.xlsx*. Where available, data means and standard deviations from replicate analyses of Chl*a*, TSS and *a*_CDOM_(440) are provided in separate files.

The most frequently used methods for Chl*a* were via solvent-based pigment extraction from filter pads followed by fluorometric (U.S. EPA 445.0) or spectrophotometric (U.S. EPA 446.0) analysis. In the majority of samples, pigments were extracted in 90% acetone with the aid of mechanical tissue grinding. Modifications of these methods included the use of 90% acetone buffered with MgCO_3_ and different approaches to support the mechanical breakdown of the algal cells. Other methods for Chl*a* followed national and international standards (DIN 38412-16:1985-12, NEN 6520, HJ 897–2017, SL88-2012 and ISO 10260:1992). Methods which included a correction for phaeophytin, a degradation product of Chl*a*^78^, are indicated by a flag (‘1’) in the data table (column ‘Phaeophytin_correction’) and the corresponding Chl*a* value is found in column ‘Chla’; where phaeophytin was not corrected for the flag is ‘0’ and Chl*a* is provided in column ‘Chl_plus_phaeo’ unless the correction for phaeophytin was not applicable as for certain fluorometric instrument setups^79^. Many investigators also used high-pressure liquid chromatography (HPLC) for Chl*a* determination and the Chl*a* value is found in column ‘Chla’. The only exception to lab-determined Chl*a* are measurements from the Thetis profiler in Lake Geneva (Switzerland) where Chl*a* associated with *R*_rs_ measurements was estimated from absorption line height at 676 nm^80^ and the linear relationship between the night-time fluorometric Chl*a* (measured by a WetLabs ECO Triplet BBFL2W) with absorption line height (average coefficient of determination: R^2^ = 0.92)^72^.

TSS concentration was measured gravimetrically by weighing the dried residue of a water sample filtered on a pre-combusted and pre-weighed filter pad. *a*_CDOM_(440) was generally quantified following Mitchell *et al*.^81^. Therefore, the optical density of water samples, typically filtered through 0.2 μm pore size polycarbonate membranes to remove particulates, was measured in a spectrophotometer and converted to absorption. Secchi depth was determined as the depth at which a disk, typically black and white of 20 or 30 cm in diameter, is no longer visible by an observer when it is lowered into the water^82,83^.

### Ancillary and metadata

Each data entry is associated with fields identifying the data contributor, cross-references to other databases, and details describing the sampling site and environmental conditions. Several categorical variables allow cursory stratification of the dataset according to water body type (lake, estuary, coastal ocean, river or other), data collection purpose (e.g., routine surface water monitoring or event-driven sampling), dominant biogeochemical water type (e.g., sediment-dominated or algal-dominated), and optical stability (e.g., low for shallow lakes, rivers and estuaries or high for deep lakes and some coastal ocean environments).

Specific characteristics of the sampling event such as geocoordinates, date and time stamps, environmental conditions (e.g., cloud cover, wind speed and wave height), and environmental settings (e.g., elevation above sea level, dominant land cover and slope) are provided where known. Several metadata fields provide cross references to details of instrumentation, measurement and processing methods for all radiometric and water quality data.

## Data Records

The GLORIA dataset is hosted at the PANGAEA Data Publisher for Earth & Environmental Science^84^. The data is contained in several comma-separated value (csv) files and a Microsoft Excel file provides keys to column names and method details (Table 1). Individual data points are identified across all files using the GLORIA_ID.

**Table 1:** Files of the GLORIA dataset and their content.

Filename	Description
*GLORIA_variables_and_methods.xlsx*	Excel file with several sheets:
	Data headers: Key to columns and units in the ancillary and metadata table (*GLORIA_meta_and_lab.csv*).
	Radiometry methods: Details of the instruments and their setups for the radiometric measurements.
	Chla methods, TSS methods, aCDOM Methods: Method details for the respective water quality measurements. Cross-referencing to data entries requires the dataset ID and methodology name from the table *GLORIA_meta_and_lab.csv*.
	References: List of references cited in this file.
*GLORIA_meta_and_lab.csv*	Ancillary information, metadata and water quality measurements associated with each *R*_rs_ spectrum. The data fields in this file are defined in *GLORIA_variables_and_methods.xlsx* sheet ‘Data headers’.
*GLORIA_Rrs.csv*	Remote sensing reflectance (*R*_rs_, sr^−1^) spectra (Rrs_350, Rrs_351, …, Rrs_900). The first column is the GLORIA sample ID.
*GLORIA_Es.csv*	Above-water downwelling irradiance (*E*_s_, W m^−2^ nm^−1^) spectra (Es_350, Es_351, …, Es_900). The first column is the GLORIA sample ID.
*GLORIA_Lw.csv*	Water-leaving radiance just above the water surface (*L*_w_, W m^−2^ sr^−1^ nm^−1^) spectra (Lw_350, Lw_351, …, Lw_900). The first column is the GLORIA sample ID.
*GLORIA_Lt.csv*	Above-water upwelling radiance (*L*_t_, W m^−2^ sr^−1^ nm^−1^) spectra (Lt_350, Lt_351, …, Lt_900). The first column is the GLORIA sample ID.
*GLORIA_Lu.csv*	Upwelling radiance just below the water surface (*L*_u_, W m^−2^ sr^−1^ nm^−1^) spectra. The first column is the GLORIA sample ID.
*GLORIA_Lsky.csv*	Sky radiance (*L*_sky_, W m^−2^ sr^−1^ nm^−1^) spectra (Lsky_350, Lsky_351, …, Lsky _900). The first column is the GLORIA sample ID.
*GLORIA_qc_flags.csv*	Quality control (QC) flags for each QC procedure described in Table 2. A value of 1 indicates that the issue has been detected.
*GLORIA_qc_ancillary*	Ancillary information for quality control flags listed in Table 3.
*GLORIA_Rrs_mean*	Mean of *R*_rs_ measurements and the number of replicates of entries where the standard deviation is available.
*GLORIA_Rrs_std*	Standard deviation of *R*_rs_ measurements. The number of replicates for the calculation of the standard deviation is provided in *GLORIA_Rrs_mean*. Standard deviation is only available for a subset of the dataset.
*GLORIA_waterqual_uncert*	Mean, standard deviation and number of replicates for water quality measurements. The replicate type is specified as ‘field’ (separate water samples taken in the field) or ‘lab’ (replicate analyses of the same water sample). This information is available for a subset of the dataset.

**Fig. 2:**
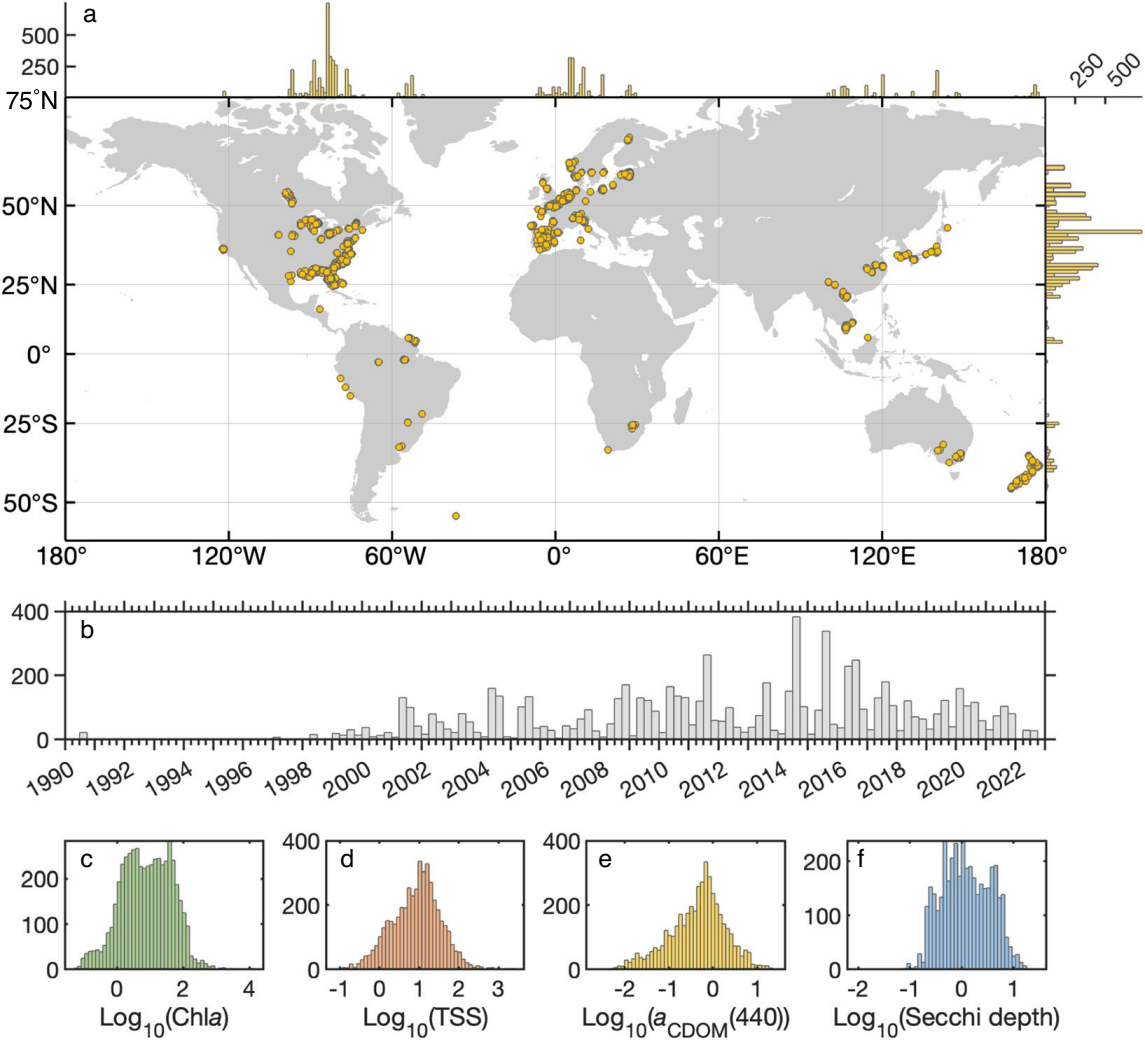
Summary of the geographical, temporal and water quality distributions of the GLORIA samples. (**a**) Dots mark the location of each sample and the histograms on the edges of the map show the longitudinal and latitudinal distributions of the dataset. (**b**) The earliest samples were collected in 1990 and the sampling effort has been steady since 2001. (**c**–**f**) Histograms of log-transformed water quality attributes illustrate the extreme range of values and their typical log-normal distributions.

**Fig. 3:**
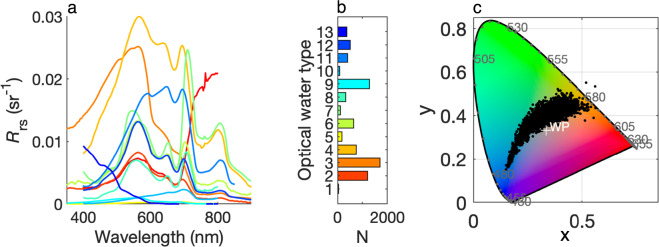
Summary of the diversity of GLORIA’s *R*_rs_ spectra. (**a**) Thirteen *R*_rs_ spectra chosen at random, one from each optical water type displayed in b. (**b**) Bar chart of the number of GLORIA spectra assigned to each optical water type from Spyrakos *et al*.^85^. (**c**) Chromaticity diagram^98^ showing the visual color derived from each GLORIA *R*_rs_ spectrum using the tristimulus weighting functions according to the Commission Internationale de l’Éclairage (CIE)^99^; WP: white point.

The 7,572 GLORIA *R*_rs_ spectra originate from 31 countries over an almost global geographical range from 67°N to 54°S and from 122°W to 178°E (Fig. 2) with the majority of samples from lakes (60%), followed by coastal waters (32%), estuaries (4%), and the remainder from rivers and other water body types. The wide range of radiometric and water quality measurements in GLORIA (Fig. 2) is consistent with the global diversity of *R*_rs_ spectral shapes with respect to optical water types^85,86^ and visual color ranges^87,88^ (Fig. 3). The range of water quality attributes is comprehensive and their frequency distributions are shown in (Fig. 2).

## Technical Validation

All data submitted for inclusion into this compilation had undergone quality control by the providers. Our curation process included detailed information recovery with them to ensure sampling, sample processing, and laboratory analysis methods are fit for purpose. Further checks on the gathered data were carried out as described below.

### Reflectance spectra

Reflectance spectra were checked for outliers and unrealistic spectral shapes using a series of quality control indicators (Table 2). By flagging, but keeping, spectra with moderate or suspected quality issues, we were able to retain a larger dataset and we advise the user to inspect the flags to evaluate the dataset for their purposes. The quality control methods are described below. Data entries with quality issues are identified by setting the corresponding quality flag to one (1) in the file *GLORIA_qc_flags.csv*.

**Table 2:** Quality control tests and associated flag names in table *GLORIA_qc_flags.csv*.

Flag name Number of cases	Description and method
Noisy_red 40	High-frequency variability, potentially instrument noise, near the red end: spectra were standardized to zero mean and unit standard deviation. A 4th order polynomial was fitted over the interval 750–900 nm. Spectra with a root-mean square error (RMSE) >0.2 were flagged. This threshold was determined using visual inspection of the distribution of RMSEs with respect to spectral shapes.
Noisy_blue 15	High-frequency variability, potentially instrument noise, near the blue end: spectra were standardized to zero mean and unit standard deviation. A 4th order polynomial was fitted over the interval 350–400 nm. Cases where root-mean square error >0.15 were flagged (threshold determined using visual inspection of the distribution of RMSEs with respect to spectral shapes).
Baseline_shift 164	Spectra shifted up are those where the minimum *R*_rs_ is 60% of its median. This percentage corresponds approximately to 1.5 times the interquartile range above the upper quartile of the baseline-percent distribution of the entire GLORIA dataset. Spectra shifted down are those with at least 20 negative values and either: • a negative linear slope in the interval 765–900 nm <−8.75 × 10^−7^ sr^−1^ nm^−1^ (the slope threshold was determined as the bound of the lower quartile) and >50% negative *R*_rs_ values in this spectral region; or • >70% negative *R*_rs_ in the interval 765 nm-900 nm; or • at least 20 negative *R*_rs_ at in the interval 350–450 nm.
Oxygen_signal 1311	Spectra where Oxygen_peak_height >0.1 (Table 3). This threshold was determined using visual inspection of the distribution of peak heights with respect to spectral shapes.
Negative_uv_slope 139	Negative slopes in the ultraviolet to blue end: The spectra were standardized to zero mean and unit standard deviation. A straight line was fitted over the interval 350–420 nm and spectra with slopes <−0.005 were flagged. This threshold was determined using visual inspection of the distribution of slope values.
QWIP_fail 278	Spectra failing a statistical quality control metric based on Apparent_visible_wavelength (Table 3). The QWIP score exceeded a value of |0.2|.
Suspect 226	Spectra identified during expert elicitation as potentially fraught with measurement problems.
Flagged 1779	A one in this column indicates the presence of at least one flag from the tests described in this table.

The first round of quality control was a procedural detection of high-frequency variability (suspected noise), baseline shifts (e.g., from suboptimal glint removal), the presence of an oxygen absorption feature near 762 nm (e.g., from sensor intercalibration issues), and negative slopes in the ultraviolet to blue part of the spectrum (e.g., from suboptimal diffuse sky radiance correction). These are the first five flags in Table 2.

Additionally, we calculated the Quality Water Index Polynomial (QWIP) score^89^. This approach was developed to identify hyperspectral aquatic reflectance data that fall outside general trends observed in a large dataset from optically deep waters. Briefly, the QWIP is a 4th order polynomial which describes a well-formed central tendency for a spectrally integrated metric (Apparent Visible Wavelength^90^, AVW) to predict a Normalized Difference Index (NDI; λ = 492, 665 nm) across a continuum of water types. For a given spectrum, the difference between the calculated NDI and that predicted by the AVW is known as the QWIP score. If a given QWIP score exceeded a prescribed deviation from the polynomial relationship, in this case |0.2|, the data was identified by the flag ‘QWIP_fail’ in the file *GLORIA_qc_flags.csv* (Table 2). AVW and the QWIP score are provided in the file *GLORIA_qc_ancillary.csv* (Table 3).

**Table 3:** Ancillary information for quality control flags in table *GLORIA_qc_ancillary.csv*.

Column name	Description and method
Oxygen_peak_height	Local maximum or minimum in *R*_rs_ near 762 nm due to absorption of light by oxygen: The spectra were standardized to zero mean and unit standard deviation. A straight line was fitted to the interval between the median values of (745–755 nm) and (775–785 nm). The maximum absolute residual of the standardized spectrum near 762 nm was recorded and is provided as a value in this column.
Apparent_visible_wavelength	Defined as the weighted harmonic mean of the visible (400–700 nm) reflectance wavelengths. This metric is used to assess the directionality and magnitude of shifts in the spectral shape of remote sensing reflectance.
QWIP_score	The QWIP score represents the difference between a calculated Normalized Difference Index (NDI; λ = 492, 665 nm) and that estimated empirically from the spectrum’s Apparent Visible Wavelength (AVW). Data producing absolute QWIP scores exceeding a value of ±0.2 have been found to exhibit spectral shapes that deviate from central tendencies typically observed in aquatic reflectance data.

On visual inspection, some spectra that passed the above criteria still appeared to have subtle problems. Further issues may be caused by instrument drift, instrument shading, stray light contamination, or errors during sky glint correction, and are often exacerbated by environmental conditions^59^. Such suspicious spectra can be recognized by experienced practitioners familiar with how inherent optical properties of surface waters vary naturally and determine reflectance through radiative transfer processes (Fig. 1)^91^. Utilizing this knowledge within the co-author community, we conducted systematic expert elicitation by randomly dividing the *R*_rs_ spectra into batches of 400 to 700 and assigning each batch to an expert for identifying suspicious looking data. The spectra that were flagged ‘Suspect’ were then evaluated by three more experts for the purpose of improving consistency across the batches from different individuals. The resulting set of suspect spectra are identified by the flag ‘Suspect’ in the file *GLORIA_qc_flags.csv* (Table 2).

### Uncertainty in *R*_rs_ from above-surface measurements by means of reconstruction with a coupled water-atmospheric radiance model

Determining the uncertainty inherent in *R*_rs_ observations is challenging because of the variable nature of illumination and water surface conditions during repeat observations. This is especially true for measurements of upwelling light made above the water surface where sun glint and reflected sky radiance contribute to *L*_t_, which applies to about 42% of the samples in GLORIA. To a large extent, spurious observations resulting from such random effects were already removed at source, such that the remaining variability is the result of various quality screening procedures and expert interpretation. However, it is possible to use models of atmospheric irradiance and bio-optical properties to model the most likely contribution of sun glint and reflected sky radiance on the *R*_rs_ observation, and thereby test the reported *R*_rs_ for physical consistency. To this end, we used the 3C algorithm^92^ to reconstruct *R*_rs_ from records where *L*_t_, *L*_sky_ and *E*_s_ were available.

3C provides a reconstruction of *R*_rs_ using nonlinear optimization of atmospheric and water optical models, allowing for a range of optical properties to solve the relationship between the upwelling radiance and downwelling irradiance provided as input. Due to the flexibility of the surface corrections, 3C is proposed to enable robust *R*_rs_ to be obtained across a wide range of measurement conditions. The resultant 3C-*R*_rs_ is expected to have reduced propagation of error from the variable spectral shape of sky reflectance and glint. This provides an advantage over methods which consider these corrections either constant, or a function of wind speed^60^, which is the case for the majority of *R*_rs_ from above-surface measurements reported in the GLORIA database (column ‘Skyglint_removal’ in *GLORIA_meta_and_lab.csv*). The difference between 3C-*R*_rs_ and the originally reported *R*_rs_ is, therefore, an approximate measure of algorithmic uncertainty. A close match between the 3C reconstruction and the originally reported *R*_rs_ provides confidence that the reported observation was physically consistent. Larger discrepancies are assumed to be associated with challenging observation conditions, resulting in suspect *L*_sky_, *L*_t_ or *E*_s_, but can also be caused by water or atmospheric properties which the model cannot reconstruct.

For this analysis, we used the 1589 spectra which included *L*_t_, *L*_sky_, *E*_s_, observation time, and geographic location, and for which the method to calculate *R*_rs_ was not already based on 3C. This analysis is also independent from the quality flagging in the previous section, so that all observations were included and the results present a worst-case scenario which best represents the algorithmic uncertainty inherent to calculating *R*_rs_, albeit without knowledge of quality control criteria applied prior to the data being reported. The 3C water optical model was configured with wide bounds for the concentration of Chl*a* (initial condition 5 mg m^−3^, range 0.01–1000 mg m^−3^) and TSS (initial condition 10 g m^−3^, range 0–1000 g m^−3^) whilst otherwise configured as detailed in Groetsch *et al*.^92^ and Jordan *et al*.^93^.

**Fig. 4:**
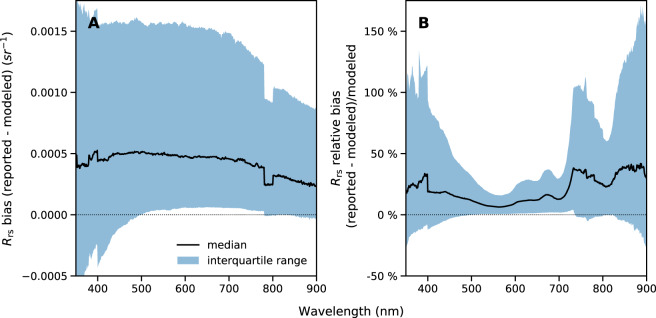
Spectral bias of reported *R*_rs_ compared with 3C-modeled *R*_rs_ from 1589 spectra for which *L*_t_, *L*_sky_ and *E*_s_ were available. (**A**) Median and interquartile (reported - modeled). (**B**) Relative bias in *R*_rs_ (reported - modeled)/modeled. Discontinuities in the bias spectrum are caused by sensors having different wavelength ranges within parts of the dataset.

The median bias between reported and 3C-*R*_rs_ was in the order of 0.0005 sr^−1^, with 3C yielding lower *R*_rs_, as should be expected because incomplete correction relying on a static correction factor for surface reflections leads to higher *R*_rs_ (Fig. 4A). Bias gradually decreased with wavelength, which suggests the reported data have been suboptimally corrected for diffuse sky radiance. There is considerable spread in the model-observation bias, in the order of 0.00004 to 0.0016 sr^−1^ for *R*_rs_(560) in the interquartile range.

In relative terms (Fig. 4B), median bias in *R*_rs_ between observed and 3C-*R*_rs_ is smallest in the green spectral range (order of 6.4%), where peak *R*_rs_ amplitude is typically observed in this dataset, and largest in the UV and NIR regions of the spectrum where *R*_rs_ is typically lower. The spread (interquartile range) in the relative bias in *R*_rs_(560) is 5–16%, but much wider in the UV and NIR range, exceeding −30% and 170%.

The largest differences in *R*_rs_ bias between reported and 3C spectra were found between contributed datasets, rather than between observation methods. The majority of datasets showed absolute relative differences in *R*_rs_(400–800) in the 0–10% range, but there are also cases where the difference exceeds 100%.

This analysis points to an overall high degree of uncertainty in the methods using above-water *L*_t_ measurements and the need for rigorous quality control by observers. For future work, we suggest adding *R*_rs_ model reconstruction as part of the data collection effort, which allows inspection of glint terms to objectively flag observations as suspect, before other quality controls are implemented. Furthermore, to support future algorithmic improvements (e.g., to elaborate bidirectional reflectance distribution functions), all component spectra and observation geometries should be included in datasets and these should be reported at the native resolution of each sensor involved to avoid convolution error when calculating *R*_rs_^94^.

### Water quality

The water quality measurements were investigated using frequency distributions to identify outliers. Separate frequency distributions were created by ‘Water_type’, a subjective classification assigned by the data contributors according to the dominant optical constituent for each water body (TSS-dominated, Chl*a*-dominated, CDOM-dominated, Chl*a* + CDOM-dominated, moderately turbid coastal, clear). Any measurements above three standard deviations from the water-type specific mean were reevaluated to ensure they were of high confidence.

## Usage Notes

### References to method details

The methods used for radiometric measurements and laboratory analyses are identified in the columns ‘Measurement_method’, ‘Chl_method’, ‘TSS_method’, and ‘aCDOM_method’ in the file *GLORIA_meta_and_lab.csv*. Associated details with references are provided in separate sheets in the file *GLORIA_variables_and_methods.xlsx*. Looking up the method for a particular measurement requires the ‘Dataset_ID’ and the method name.

### Quality flags

Each *R*_rs_ measurement is associated with quality flags (file *GLORIA_qc_flags.csv*). The quality flags are binary and indicate the presence (‘1’) or absence (‘0’) of the quality issue described in Table 2. Missing values indicate that the flag could not be determined because the spectrum did not include the required wavelength range. Some numerical values generated during the quality control are provided in the file *GLORIA_qc_ancillary.csv* (Table 3).

### Cross-references to other datasets

Some of the data in GLORIA is part of other data publications, or is also included in the community repositories SeaBASS^33^ and/or LIMNADES. The columns ‘SeaBASS_ID’, ‘LIMNADES_ID’, and ‘LIMNADES_UID’ in the data table (*GLORIA_meta_and_lab.csv*) provide identifiers used in the respective datasets to facilitate cross referencing entries, for example for the avoidance of duplicates. Other references to prior publication of the data are provided in the ‘Comments’ column in *GLORIA_meta_and_lab.csv* in the form of a digital object identifier (DOI).

## Data Availability

The code to conduct the quality control flagging described in the section *Technical validation* is written in R^95^ and available on Zenodo^96^. The 3C code is available at https://gitlab.com/pgroetsch/Rrs_model_3C. The code for QWIP is on Zenodo^97^.
