# Author Correction: GLORIA - A globally representative hyperspectral *in situ* dataset for optical sensing of water quality

**DOI:** 10.1038/s41597-023-02069-3

**Published:** 2023-04-06

**Authors:** Moritz K. Lehmann, Daniela Gurlin, Nima Pahlevan, Krista Alikas, Ted Conroy, Janet Anstee, Sundarabalan V. Balasubramanian, Cláudio C. F. Barbosa, Caren Binding, Astrid Bracher, Mariano Bresciani, Ashley Burtner, Zhigang Cao, Arnold G. Dekker, Courtney Di Vittorio, Nathan Drayson, Reagan M. Errera, Virginia Fernandez, Dariusz Ficek, Cédric G. Fichot, Peter Gege, Claudia Giardino, Anatoly A. Gitelson, Steven R. Greb, Hayden Henderson, Hiroto Higa, Abolfazl Irani Rahaghi, Cédric Jamet, Dalin Jiang, Thomas Jordan, Kersti Kangro, Jeremy A. Kravitz, Arne S. Kristoffersen, Raphael Kudela, Lin Li, Martin Ligi, Hubert Loisel, Steven Lohrenz, Ronghua Ma, Daniel A. Maciel, Tim J. Malthus, Bunkei Matsushita, Mark Matthews, Camille Minaudo, Deepak R. Mishra, Sachidananda Mishra, Tim Moore, Wesley J. Moses, Hà Nguyễn, Evlyn M. L. M. Novo, Stéfani Novoa, Daniel Odermatt, David M. O’Donnell, Leif G. Olmanson, Michael Ondrusek, Natascha Oppelt, Sylvain Ouillon, Waterloo Pereira Filho, Stefan Plattner, Antonio Ruiz Verdú, Salem I. Salem, John F. Schalles, Stefan G. H. Simis, Eko Siswanto, Brandon Smith, Ian Somlai-Schweiger, Mariana A. Soppa, Evangelos Spyrakos, Elinor Tessin, Hendrik J. van der Woerd, Andrea Vander Woude, Ryan A. Vandermeulen, Vincent Vantrepotte, Marcel R. Wernand, Mortimer Werther, Kyana Young, Linwei Yue

**Affiliations:** 1 Xerra Earth Observation Institute, PO Box 400, Alexandra, 9340 New Zealand; 2 School of Science, University of Waikato, Private Bag 3105, Hamilton, 3240 New Zealand; 3 Wisconsin Department of Natural Resources, Bureau of Water Quality, 101 S Webster Street, Madison, WI 53707 USA; 4 Science Systems and Applications, Inc. (SSAI), Lanham, MD USA; 5 NASA Goddard Space Flight Center, Greenbelt, MD USA; 6 Tartu Observatory of the University of Tartu, Tartumaa, 61602 Estonia; 7 Coasts and Oceans Systems Program (COS), CSIRO Environment Business Unit, Acton, ACT 2601 Australia; 8 GeoSensing and Imaging Consultancy, Trivandrum, Kerala India; 9 Instrumentation Lab for Aquatic Systems (LabISA), National Institute for Space Research (INPE), São José dos Campos, Brazil; 10 Environment and Climate Change Canada, Burlington, ON Canada; 11 Phytooptics Group, Physical Oceanography of Polar Seas, Climate Sciences, Alfred Wegener Institute, Helmholtz Centre for Polar and Marine Research, Bremerhaven, Germany; 12 Department of Physics and Electrical Engineering, Institute of Environmental Physics, University of Bremen, Bremen, Germany; 13 National Research Council of Italy, Institute for Electromagnetic Sensing of the Environment, CNR-IREA, Milano, Italy; 14 Cooperative Institute for Great Lakes Research, University of Michigan, 4840 South State Road, Ann Arbor, MI 48108 USA; 15 Nanjing Institute of Geography and Limnology, Chinese Academy of Sciences, Nanjing, 210008 China; 16 SatDek Pty Ltd, 99 Read Rd, Sutton, NSW 2620 Australia; 17 Wake Forest University, Engineering, 455 Vine Street, Winston-Salem, NC 27101 USA; 18 NOAA Great Lakes Environmental Research Laboratory, Ann Arbor, MI USA; 19 Department of Geography, Universidad de la República, Montevideo, Uruguay; 20 Institute of Biology and Earth Sciences, Pomeranian University, Arciszewskiego 22, 76-200 Slupsk, Poland; 21 Department of Earth and Environment, Boston University, Boston, MA USA; 22 German Aerospace Center (DLR), Remote Sensing Technology Institute, Wessling, Germany; 23 University of Nebraska-Lincoln, School of Natural Resources, 3310 Holdrege Street, Lincoln, NE 68503 USA; 24 University of Wisconsin-Madison, Aquatic Sciences Center, 1975 Willow Drive, Madison, WI 53706 USA; 25 Michigan Technological University, Great Lakes Research Center, 100 Phoenix Drive, Houghton, MI 49931 USA; 26 Faculty of Urban Innovation, Yokohama National University, Tokiwadai 79-5, Hodogaya, Yokohama, Kanagawa Japan; 27 Swiss Federal Institute of Aquatic Science and Technology, Department of Surface Waters – Research and Management, Dübendorf, Switzerland; 28 Université du Littoral Côte d’Opale, CNRS, Univ. Lille, IRD, UMR 8187 - LOG - Laboratoire d’Océanologie et de Géosciences, F-62930 Wimereux, France; 29 Earth and Planetary Observation Sciences (EPOS), Biological and Environmental Sciences, Faculty of Natural Sciences, University of Stirling, Stirling, UK; 30 Plymouth Marine Laboratory, Plymouth, PL1 3DH UK; 31 NASA Ames Research Center, Moffett Field, CA USA; 32 Department of Physics and Technology, University of Bergen, Bergen, Norway; 33 University of California-Santa Cruz, Ocean Sciences Department, Institute of Marine Sciences, 1156 High Street, Santa Cruz, CA 95064 USA; 34 Department of Earth Sciences, Indiana University-Purdue University, Indianapolis, IN USA; 35 University of Massachusetts-Dartmouth, School for Marine Science and Technology West, 706 South Rodney French Blvd., New Bedford, MA 02744 USA; 36 Coasts and Oceans Systems Program (COS), CSIRO Environment Business Unit, Ecosciences Precinct, 41 Boggo Road, Dutton Park, QLD 4102 Australia; 37 Faculty of Life and Environmental Sciences, University of Tsukuba, Ibaraki, Japan; 38 CyanoLakes (Pty) Ltd, Sydney, 2126 Australia; 39 Departament de Biologia Evolutiva, Ecologia i Ciències Ambientals, Facultat de Biologia, Universitat de Barcelona, Av. Diagonal 643, 08028 Barcelona, Spain; 40 Department of Geography, University of Georgia, Athens, GA 30602 USA; 41 National Centers for Coastal Ocean Science, National Oceanic and Atmospheric Administration, 1305 East-West Hwy, Silver Spring, MD 20910 USA; 42 Harbor Branch Oceanographic Institute, Florida Atlantic University, Fort Pierce, FL USA; 43 U.S. Naval Research Laboratory, 4555 Overlook Ave SW, Washington, DC 20375 USA; 44 Faculty of Geology, VNU University of Science, Ha Noi, Vietnam; 45 Royal Netherlands Institute for Sea Research, Physical Oceanography, Marine Optics & Remote Sensing, Den Burg, Texel, Netherlands; 46 Upstate Freshwater Institute, Syracuse, NY USA; 47 Department of Forest Resources, University of Minnesota, St. Paul, MN USA; 48 NOAA Center for Satellite Applications and Research, College Park, MD USA; 49 Earth Observation and Modelling, Kiel University, Department of Geography, 24118 Kiel, Germany; 50 UMR LEGOS, University of Toulouse, IRD, CNES, CNRS, UPS, 14 Avenue Edouard Belin, 31400 Toulouse, France; 51 Department Water-Environment-Oceanography, University of Science and Technology of Hanoi (USTH), Vietnamese Academy of Science and Technology (VAST), 18 Hoang Quoc Viet, Hanoi, 100000 Vietnam; 52 Department of Geosciences, Federal University of Santa Maria, Av. Roraima, 1000, 97105-900 Santa Maria, Rio Grande do Sul Brazil; 53 Laboratory for Earth Observation, University of Valencia, Catedrático Agustín Escardino 9, Paterna (Valencia), 46980 Spain; 54 Faculty of Engineering, Kyoto University of Advanced Science (KUAS), 18 Yamanouchi Gotanda, Ukyo, Kyoto, Japan; 55 Creighton University, Department of Biology, Omaha, NE 68178 USA; 56 Japan Agency for Marine-Earth Science and Technology (JAMSTEC), Showa-machi 3173-25, Yokohama, Kanagawa 2360001 Japan; 57 Department of Water & Climate Risk, Institute for Environmental Studies (IVM), Vrije Universiteit, Amsterdam, Netherlands; 58 Ocean Ecology Laboratory, NASA Goddard Space Flight Center, Greenbelt, MD USA; 59 China University of Geosciences, School of Geography and Information Engineering, Wuhan, China

**Keywords:** Limnology, Environmental impact

Correction to: *Scientific Data* 10.1038/s41597-023-01973-y, published online 16 February 2023.

An author of the paper was omitted in the original version (Ted Conroy, University of Waikato, New Zealand). This has been corrected in the pdf and HTML versions of the paper, and the associated metadata.

